# Alcohol and Suicide: Neurobiological and Clinical Aspects

**DOI:** 10.1100/tsw.2006.146

**Published:** 2006-06-21

**Authors:** Leo Sher

**Affiliations:** Division of Neuroscience, Department of Psychiatry, Columbia University, New York, USA

**Keywords:** alcohol, suicide, depression, aggression, serotonin, dopamine, brain damage, United States

## Abstract

Alcohol, primarily in the form of ethyl alcohol (ethanol), has occupied an important place in the history of humankind for at least 8,000 years. In most Western societies, at least 90% of people consume alcohol at some time during their lives, and 30% or more of drinkers develop alcohol-related problems. Severe alcohol-related life impairment, alcohol dependence (alcoholism), is observed at some time during their lives in about 10% of men and 3—5% of women. An additional 5—10% of each sex develops persistent, but less intense, problems that are diagnosed as alcohol abuse. It this review, neurobiological aspects of suicidal behavior in alcoholism is discussed. In individuals with comorbid depression and alcoholism, greater serotonergic impairment may be associated with higher risk of completed suicide. Dopaminergic dysfunction may play an important role in the pathophysiology of suicidal behavior in alcoholism. Brain damage and neurobehavioral deficits are associated with alcohol use disorders and may contribute to suicidal behavior in persons with alcohol dependence or abuse. Aggression/impulsivity and alcoholism severity affect risk for suicide among individuals with alcoholism. Major depressive episodes and stressful life events particularly, partner-relationship disruptions, may precipitate suicidal behavior in individuals with alcohol use disorders. Alcohol misuse and psychosocial adversity can combine to increase stress on the person, and, thereby, potentially, increase the risk for suicidal behavior. The management of suicidal patients with alcohol use disorders is also discussed. It is to be hoped that the efforts of clinicians will reduce morbidity and mortality associated with alcohol misuse.

## INTRODUCTION

Alcohol, primarily in the form of ethyl alcohol (ethanol), has occupied an important place in the history of humankind for at least 8,000 years. In most Western societies, at least 90% of people consume alcohol at some time during their lives, and 30% or more of drinkers develop alcohol-related life problems[1,2]. Severe alcohol-related life impairment, alcohol dependence (alcoholism), is observed at some time during their lives in 10% of men and 3–5% of women, with an additional 5–10% of each gender developing persistent, but less intense, problems that are diagnosed as abuse. Alcohol dependence is a maladaptive pattern of alcohol use, leading to clinically significant impairment or distress, as manifested by three (or more) of the following, occurring at any time in the same 12-month period: (1) tolerance; (2) withdrawal; (3) alcohol is often taken in larger amounts or over a longer period than was intended; (4) there is a persistent desire or unsuccessful efforts to cut down or control alcohol use; (5) a great deal of time is spent in activities necessary to obtain alcohol, use alcohol, or recover from its effects; (6) important social, occupational, or recreational activities are given up or reduced because of alcohol use; (7) alcohol use is continued despite knowledge of having a persistent or recurrent physical or psychological problem that is likely to have been caused or exacerbated by alcohol[2]. Alcohol abuse is a maladaptive pattern of alcohol use leading to clinically significant impairment or distress, as manifested by one (or more) of the following, occurring within a 12-month period: (1) recurrent alcohol use resulting in a failure to fulfill major role obligations at work, school, or home; (2) recurrent alcohol use in situations in which it is physically hazardous; (3) recurrent alcohol-related legal problems; (4) continued alcohol use despite having persistent or recurrent social or interpersonal problems caused or exacerbated by the effects of alcohol[2].

Ethanol is a weakly charged molecule that moves easily through cell membranes, rapidly equilibrating between blood and tissues[3]. The level of alcohol in the blood is expressed as milligrams or grams of ethanol per deciliter (e.g., 100 mg/dl or 0.10 g/dl); a level of 0.02 to 0.03 results from the ingestion of one to two typical drinks. In round figures, 340 ml (12 oz) of beer, 115 ml (4 oz) of nonfortified wine, and 43 ml (1.5 oz) (a shot) of 80-proof beverage each contain ~10 to 15 g of ethanol; 0.5 l (1 pint) of 86-proof beverage contains ~160 g (about 16 standard drinks), and 1 l of wine contains ~80 g of ethanol.

Women appear to be more vulnerable than men to many adverse consequences of alcohol use[4,5]. Epidemiologic evidence clearly indicates that the adverse consequences of alcohol consumption, including severe liver disease, such as alcoholic cirrhosis, develop more quickly and require lower levels of alcohol exposure for females than for males. In the U.S., the cost to society of alcohol misuse is about 200 billion dollars every year[6]. These costs include expenditures on alcohol-related problems and opportunities that are lost because of alcohol.

Alcohol dependence and abuse are often associated with dependence on, or abuse of, other substances (e.g., cannabis; cocaine; heroin; amphetamines; the sedatives, hypnotics, and anxiolytics; and nicotine)[2,7]. Alcohol may be used to alleviate the unwanted effects of these other substances or to substitute for them when they are not available. Symptoms of depression, anxiety, and insomnia frequently accompany alcohol dependence and often precede it.

## AN HISTORICAL PERSPECTIVE

Physicians have been interested in the relationship between alcohol and suicide for many decades. A little over 150 years ago, in a book on alcoholism, Dr. Charles Wilson[8] of Edinburgh wrote: "… should [the alcoholic] lift his hands against his life, armed with an instrument more promptly fatal, it is by a kind of double-suicide that the last act is perpetrated. The more gradual self-destruction, which simulates disease, is crowned by the rapid catastrophe which kills by violence. It is the character of the act which is changed, but not its essence." Then 90 years later, Dahlgren[9], whose book was a review of the European literature on suicide and attempted suicide, concluded that the majority of authors have ascribed a comparatively great importance to alcoholism as a suicide-eliciting factor.

## EPIDEMIOLOGY

Alcohol use disorders are an important risk factor for suicidal behavior. It was suggested that lifetime mortality due to suicide in alcohol dependence is as high as 18%[10]. However, Murphy and Wetzel[11] reviewed the epidemiological literature and found that the lifetime risk of suicide among individuals with alcohol dependence treated in out-patient and in-patient settings was 2.2 and 3.4%, respectively. Nonetheless, individuals with alcoholism have a 60–120 times greater suicide risk than the nonpsychiatrically ill population. High rates of suicide attempts among individuals with alcohol use disorders have also been reported[12,13,14,15]. For example, in an urban community in the U.S., 24% of subjects with alcoholism attempted suicide, as compared with 5% with other psychiatric diagnoses[14]. Of a sample of depressed subjects with alcoholism who were hospitalized, 40% had attempted suicide in the prior week and 70% had attempted suicide at some point in their lives[15]. Depressed subjects with a history of alcoholism have higher current suicide ideation scale scores compared with depressed subjects without a history of alcoholism[16]. These data indicate that a lifetime diagnosis of alcoholism is a major risk factor for attempted or completed suicide. The large population of individuals with alcohol use disorders, the relative frequency of suicides and suicide-related behaviors in this population, and the devastating effects of attempted and completed suicides on individuals, families, and society make this a topic that requires our attention.

## NEUROBIOLOGICAL ASPECTS

### Serotonin

Multiple lines of evidence suggest that lower serotonin activity is tied to increased aggression/impulsivity, which in turn is presumed to enhance the probability of suicidal behavior[17,18]. Low cerebrospinal fluid (CSF) 5-hydroxyindolacetic acid (5-HIAA) has been reported in suicide attempters with major depression, schizophrenia, and personality disorders as compared with people who do not attempt suicide, but have the same psychiatric diagnosis[17,18,19]. The levels of 5-HIAA in CSF taken from abstinent individuals with a history of alcohol dependence of both sexes were shown to be lower than in controls[20]. Moreover, impulsive offenders with alcohol dependence had lower CSF 5-HIAA levels than nonimpulsive offenders with alcohol dependence[21]. High-lethality, depressed suicide attempters with comorbid alcoholism have lower CSF 5-HIAA levels compared with low-lethality, depressed suicide attempters with comorbid alcoholism[22]. In subjects with comorbid depression and alcoholism, greater serotonergic impairment may be associated with higher risk of completed suicide.

### Dopamine

Chen et al.[23] suggest that pathological aggression may be related to genetically determined abnormalities in the dopaminergic system. Suicidal behavior may be regarded as self-directed pathological aggression. Several lines of evidence suggest that the dopamine system is involved in the pathophysiology of suicidal behavior. Lower levels of CSF homovanillic acid (HVA) have been found in depressed patients with a history of either violent or nonviolent suicide attempts than in controls[24]. In our recent study, we compared CSF HVA levels in depressed suicide attempters without comorbid Axis II disorders, depressed nonattempters without comorbid Axis II disorders, and normal controls[25]. Depressed suicide attempters had lower CSF HVA levels compared to depressed nonattempters and to controls. There was no difference in CSF HVA levels between depressed nonattempters and controls. Several studies that did not involve CSF HVA measures also suggested that the dopaminergic system may be involved in the neurobiology of suicidal behavior. For example, Pitchot et al.[26] studied the growth hormone (GH) response to apomorphine, a selective dopaminergic agonist, in depressed patients with and without history of suicide attempts and found that patients with a history of suicidal behavior exhibited a significantly lower GH response to apomorphine than patients who never attempted suicide. More recent reports from the same group confirmed their earlier finding. It has also been observed that dopamine abnormalities are associated with impulsivity, emotional dysregulation, and alcohol use disorders[27]. For example, a recent neuroimaging study suggests that alcoholism is associated with blunted dopamine transmission in the ventral striatum[28]. Alcoholism is also associated with high aggression, impulsivity, and suicidal behavior[29]. Therefore, it is reasonable to suggest that genetically determined dopaminergic dysfunction may play an important role in the pathophysiology of suicidal behavior in alcoholism.

### Brain Damage

Alcohol can damage the brain in many ways[30,31]. The brain is vulnerable to the toxic effects of alcohol itself and can be affected by alcohol-related damage to other organs, including the liver, pancreas, and heart. The risk of alcohol-induced brain damage and related neurobehavioral deficits varies from person to person and is influenced by factors such as age, gender, drinking history, and nutrition. In the U.S., up to 2 million individuals with alcoholism have permanent and debilitating conditions that require lifetime custodial care. Examples of such conditions include alcohol-induced persisting amnesic disorder (also called Wernicke-Korsakoff syndrome) and dementia, which seriously affects many mental functions in addition to memory (e.g., language, reasoning, and problem-solving abilities). Brain damage and neurobehavioral deficits are associated with suicidal behavior[32,33].

## CLINICAL ASPECTS

### Chronic Effects of Alcohol

A model of suicidal behavior among subjects with alcoholism has recently been proposed[34]. Predisposing factors that are presumed to increase (moderate) risk for suicide among individuals with alcoholism are aggression/impulsivity and alcoholism severity, which represent predominantly externalizing constructs, and negative affect and hopelessness, which represent predominantly internalizing constructs. Major depressive episodes and stressful life events – particularly interpersonal difficulties – are conceptualized as precipitating factors. This model is consistent with the stress-diathesis model of suicidal behavior[35,36]. Partner-relationship disruptions are strongly associated with suicidal behavior in individuals with alcohol dependence. Alcohol misuse and psychosocial adversity can combine to increase stress on the person, and, thereby, potentially, increase the risk for suicidal behavior. There may be a broad-based social characteristic related to social stress that is associated with high rates of a variety of stress-related behaviors, including heavy alcohol use, divorce, and suicide[37]. The combination of depression and alcohol dependence often leads to suicidal behavior[16,29,38,39]. Treatment for this fatal combination remains poor, and there is no evidence-based guidance as to the choice of biological and/or psychological treatments for this population when suicide is a prime concern. Future studies are necessary to determine what interventions may reduce suicidal behavior in individuals with comorbid depression and alcohol dependence.

### Acute Effects of Alcohol

The acute effects of alcohol use act as important risk factors for attempted or completed suicide among individuals with and without alcoholism[40,41]. Mechanisms responsible for alcohol's ability to increase the risk for suicidal behavior include alcohol's ability to increase aggressiveness, increase psychological distress, propel suicidal ideation into action through suicide-specific alcohol expectancies, and constrict cognition that impairs the generation and implementation of alternative coping strategies. The association between acute alcohol use and aggression has been documented by both correlational and experimental studies[42,43].

## MANAGEMENT

Patients with alcoholism who report a suicide plan or intent must be hospitalized and closely observed[29,44]. A person with alcoholism and symptoms of depression severe enough to be suicidal requires in-patient hospitalization for at least several days, until the suicidal ideation disappears, even if it is a temporary alcohol-induced depression. Hospitalization should also be considered if an individual with alcoholism exhibits severe agitation, psychotic symptoms, thought disorder, or prominent impulsivity; reports feeling of hopelessness; and/or has a history of suicide attempt. When the suicidal symptoms have abated, discharge from in-patient care may be considered. Before discharge, the family of the patient should be advised to remove all items of danger from the home, such as guns and poisons, and to provide close observation of the patient after discharge. An appropriate referral to an alcohol abuse treatment program should be given.

In summary, alcohol abuse and dependence are associated with depression and suicidal behavior as well as with medical and neurological problems. It is to be hoped that the efforts of clinicians will reduce morbidity and mortality associated with alcohol misuse.

## BIOSKETCH

**Leo Sher, MD** is a psychiatrist with a background in internal medicine. His areas of research and clinical expertise include neurobiology, diagnosis, and treatment of mood, anxiety, and personality disorders, alcoholism, and suicidal behavior. He graduated Summa Cum Laude from the Ukrainian National

Medical University in Kiev, and did his residency in psychiatry at the Albert Einstein College of Medicine Program at Long Island Jewish Medical Center in New York and the National Institute of Mental Health Program in Bethesda, MD. He completed a Research Fellowship at the National Institute of Mental Health in Bethesda, MD. Currently, Dr. Sher is Assistant Clinical Professor of Psychiatry at Columbia University and Research Psychiatrist in the Department of Neuroscience at the New York State Psychiatric Institute in New York City.

He has authored more than 250 scientific publications, has been a reviewer for numerous medical journals, recipient of several awards, and the first researcher in North America who introduced the use of the combined dexamethasone suppression/corticotropin-releasing hormone stimulation test for psychiatric purposes. E-mail: LS2003@columbia.edu Website: http://asp.cumc.columbia.edu/facdb/profile_list.asp?uni=ls2003&DepAffil=Psychiatry
